# Association of toll-like receptors polymorphism and intrauterine transmission of cytomegalovirus

**DOI:** 10.1371/journal.pone.0189921

**Published:** 2017-12-21

**Authors:** Yifat Eldar-Yedidia, Miriam Hillel, Amitay Cohen, Maskit Bar-Meir, Yossi Freier-Dror, Yechiel Schlesinger

**Affiliations:** 1 Research Laboratory of Infectious Diseases, Shaare Zedek Medical Center, affiliated to the Hebrew University Medical School, Jerusalem, Israel; 2 Hadassah- Hebrew University Medical School, Jerusalem, Israel; 3 Department of Pediatrics, Shaare Zedek Medical Center, Jerusalem, Israel; 4 Mashav Applied Research, Jerusalem, Israel; Stellenbosch University, SOUTH AFRICA

## Abstract

**Background:**

Congenital Cytomegalovirus (CMV) is a very common intrauterine infection which can cause severe developmental disabilities. Transmission of the virus to the fetus occurs in only 40% of primarily infected women. The probability of intrauterine transmission is higher when infection occurs during the second trimester of pregnancy than in the first trimester. The Toll-like receptors (TLRs) protein family plays a key role in both innate immune response to CMV infections and in normal pregnancy. Specific single nucleotide polymorphisms (SNPs) in TLRs can affect CMV infections and maternal–fetal interface expression. Therefore, TLR SNPs could be involved in intrauterine transmission determination.

**Study aim:**

To establish a correlation between TLR2 (rs4696480, rs3804100, rs1898830), TLR3 (rs3775291) and TLR7(rs179008) SNPs with CMV intrauterine transmission during the first and second trimester.

**Methods:**

SNPs of 83 pregnant women with primary CMV were analyzed by Real-Time PCR and PCR-RFLP assay and compared to intrauterine transmission state.

**Results:**

Women bearing the GG genotype in the rs1898830 TLR2 SNP who were infected with CMV during the second trimester did not transmit the virus to the fetus. Likewise, in the co-dominant or recessive models of this SNP, a significant association was found between the genotypes and CMV intrauterine transmission. In all cohort women or in women infected during the first trimester, no such associations were found between the tested SNPs and intrauterine transmission of the virus.

**Conclusion:**

Women bearing the GG genotype in the rs1898830 SNP, who are infected with CMV during the second trimester of pregnancy, have a low likelihood of transmitting the virus to the fetus.

## Introduction

Cytomegalovirus (CMV) is the most common cause of congenital infection in the developed world, and can cause a variety of long-term disabilities including mental, hearing and visual impairments [1–3]. These severe disabilities threaten more children than well-known childhood maladies such as Down syndrome or fetal alcohol syndrome [4,5]. The general maternal-fetal transmission rate of CMV is about 40%. However, the intrauterine transmission rates change along the pregnancy, and they are about 30%, 38% and 72% during the first, second and third trimesters, respectively [6].

Toll-like receptors (TLRs) play a crucial role in non-specific immunity and recognition of highly conserved structures of pathogens [7]. Pathogen structures activate an intracellular signaling cascade mediated by TLRs, causing induction of antimicrobial genes and inflammatory cytokines. This early response to infection is crucial until the pathogen-specific adaptive arm of the immune response is established [7].

The TLR protein family was shown to be involved in normal and pathological pregnancy process such as preterm labor, preeclampsia or IUGR (reviewed by [8]). Other studies have shown that various TLRs play a role in non-specific immunity to CMV infection course (reviewed by [8]).

Single nucleotide polymorphisms (SNPs) within some TLR genes have been shown to modify the immune response to CMV infections [9–11] and to affect the maternal–fetal interface (reviewed by [8]). Therefore, we hypothesized that genetic variation within these genes plays a role in determining CMV intrauterine transmission.

To test this hypothesis, we selected five TLRs SNPs that were considered to be important for CMV infection. Two TLR-2 SNPs (rs3804100: T>C and rs1898830:A>G) have been shown in a Japanese infant population to have some association with congenital CMV infection [12]. Two additional SNPs are relevant to the cytokine response; the TLR2 promoter variant (rs4696480: T>A) has been shown to significantly enhance Th2-cytokines and TNF-α secretion in children of mothers with maternal atopy [13]. Another SNP, TLR3 variant (rs3775291: C>T) yielding an amino-acid change (L to F), is associated with reduced IFN-γ and TNF-α secretion in response to stimulation with the TLR3 ligand or CMV [11]. One of the SNPs in TLR7 (rs179008: A>T) induces the change of glutamine to leucine in position 11 and was shown to be associated with the level of antibodies to the gB envelop protein following vaccination with CMV gB[9]. Other TLRs SNPs which have been shown to play a role in CMV infection were not included in this study for various reasons. For example, TLR2 rs5743708 [14] that was shown to modify CMV disease in liver transplant recipients was excluded due to minor genotype frequency (MAF) lower than 5% in the CEU population.

In the present study we sought an association between these 5 TLR's SNPs and intrauterine transmission in 83 pregnant women with primary CMV infection. The genotype of rs4696480 and rs3804100 in TLR2 and rs179008 in TLR7 were determined by RFLP (Restriction fragment length polymorphism). The genotypes of rs1898830 in TLR2 and rs3775291 in TLR3 were determined by RT-PCR. We found that in women infected with CMV during the second trimester, the GG genotype of SNP rs1898830 is significantly associated with lack of CMV intrauterine transmission.

## Materials and methods

### Human subjects

This study was approved by the local ethics committee of Shaare-Zedek Medical Center and written informed consent was obtained from each participating woman. The study was performed according to Good Clinical Practice (GCP) guidelines.

Samples were collected from 83 pregnant women who were diagnosed with primary CMV infection. The diagnosis was made by one of the following serological findings: CMV-specific IgG seroconversion or the presence of low avidity IgG antibodies or CMV- specific IgM with no previous IgG antibodies. The timing of primary infection was determined by the time of seroconversion and/or analysis of the increment of IgG avidity and/or by clinical symptoms [15]. Intrauterine CMV transmission was determined by detection of viral DNA by real-time PCR, either in amniotic fluid or in the newborn’s urine. The analysis of these specimens was performed by the treating physicians in their respective medical centers throughout Israel. Transmission results were reported to us.

### SNP analysis

Genomic and viral DNA was extracted from 200μl blood sample using a DNeasy Blood & Tissue Kit (QIAGEN, Germany) according to the manufacturer’s instructions. Extracted DNA was diluted in 100μl of elution buffer and stored at -80°C until molecular analysis was performed.

Targeted SNPs were selected according to their role in CMV infection or cytokine secretion. All selected SNPs have MAF > 5% in the CEU (Utah Residents with Northern and Western Ancestry) population. The frequencies of genotypes of each SNP in the CEU population were obtained from the "1000 Genomes" Project (http://www.1000genomes.org/).

The genotyping of TLR2 rs1898830 and TLR3 rs3775291 was performed by Taqman SNP assays according to the manufacturer’s guidelines (Applied Biosystems, CA). The genotyping of rs4696480 and rs3804100 from TLR2 and rs179008 from TLR7 was performed by RFLP analysis.

The fragments containing the polymorphic sites of the tested SNP were amplified by PCR Taq Master Mix (Lamda Biotech, USA). The primers: F- GTT CTG GAG TCT GGG AAG TC and R-AAT GTT ATC ACC AAG GGA GCA G were used for rs4696480 and gave a product size of 171 bp. The primers: F- AACCGGAGAGACTTTGCTCA and R- GCGGCAAATTCAAAGAAAAT were used for rs3804100 and gave a 221bp product fragment. For rs179008 we used the primers: F- AATGCTGCTTCTACCCTCTCG and R-TGTCAAATGCTTGTCTGTGCAGTC, which gave a product size of 426bp.

The PCR amplification method was as follows: 3 min at 94°C followed by 40 cycles each of 30 seconds of denaturation at 95°C, 30 seconds annealing (rs4696480 at 67°C, rs3804100 at 60°C and rs179008 at 65°C) and 20 seconds elongation at 72°C.

PCR products were digested for 1 hour at 37°C with Hpy188III (New England Biolabs, USA) for rs4696480, with HpyCH4III (New England Biolabs, USA) for rs3804100 and with ApoI-Fast Digest (Thermo Scientific, USA) for rs179008. The DNA fragments were analyzed on 2% agarose gel and the SNP variation was determined by band patterns.

### Statistical analysis

To analyze the association between the tested SNPs and intrauterine transmission of CMV, we compared the women who transmitted the virus to the fetus with those who did not. Statistical analysis was based on SNPStats software (http://bioinfo.iconcologia.net/snpstats/start.htm) [16].

First, the association between genetic polymorphisms and CMV transmission was tested by Hardy-Weinberg (H.W.) equilibrium. The observed allele frequencies were compared to the expected frequencies under the assumption of independence, using Chi-square or Fisher's exact tests.

To assess the association between SNPs and CMV intrauterine transmission, the polymorphism was described as a categorical variable with one level for each possible genotype. The homozygous genotype of the prevalent allele was used as a reference group. A Chi-square test with Yates continuity correction was conducted to test the significance of the associations. Significance was defined as a two tailed *p* < .05, ORs and 95% CI were also calculated.

## Results

The study cohort included 83 Israeli pregnant women with primary CMV infection. Their average age was 29.6 (range 21–40). CMV transmission occurred in 33 out of 83 pregnancies (39.7%). Consistent with current knowledge [6,17,18], when CMV infection onset was in the first trimester of pregnancy, transmission rate (16/ 52 = 30%) was lower than that of women who were infected in the second trimester (17/31 = 54%). Other clinical characteristics of these women are presented in Table 1.

**Table 1:** Clinical characteristics of CMV-infected pregnant women.

	Transmitters Mean ±SD (n = 33)	Non-transmitters Mean±SD (n = 50)	*P*-value
**Age**	30.3± 3.7 (22–37)	29.2± 4.7 (21–40)	NS
**Gestational age at onset of CMV infection (weeks)**	13.3± 6.9 (1–24)	9.7± 6.1 (1–24)	0.017
**Gestational age at blood collection (weeks)**	20.4± 6.3 (10–32)	16.4± 5.8 (8–30)	0.004
**Time interval between the infection onset and blood collection (weeks)**	7.2± 3.9 (3–20)	6.7±2.2 (2–12.5)	NS

NS = not significant

### Association between genotype frequencies and CMV transmission

The genotype variation frequencies of the selected SNPs in our cohort were: rs4696480: 0.42, rs3804100: 0.09, rs1898830: 0.38, rs377529: 0.26 and rs179008: 0.23. The frequencies of all SNPs genotypes were in Hardy-Weinberg equilibrium for both transmitters and non-transmitters women (Table 2).

**Table 2:** The genotype frequencies of TLR2 (rs4696480, rs3804100, rs1898830), TLR3(rs3775291) and TLR7(rs179008) SNPs, in transmitters and non-transmitters, were in H-W equilibrium.

		P-value^a^
**rs4696480**	All subjects	0.5
	transmission	0.39
	No transmission	1
**rs3804100**	All subjects	0.19
	transmission	0.33
	No transmission	0.38
**rs1898830**	All subjects	0.82
	transmission	1
	No transmission	0.71
**rs3775291**	All subjects	0.78
	transmission	1
	No transmission	0.7
**rs179008**	All subjects	0.34
	transmission	0.22
	No transmission	0.65

^a^P≤0.05 considered as significant.

For each SNP the distribution of the alleles in relation to transmission status was analyzed assuming three genotype models; dominant, recessive, or co-dominant.

Analysis of genotype frequencies in relation to transmission was performed separately on women with first and second trimester onset of infection and on the whole cohort. When analysis was done on all women (S1 Table) or was limited to women who were infected in the first trimester (Table 3), no significant associations were found between these SNPs genotype frequencies and intrauterine transmission.

**Table 3:** The distribution of genotype frequencies of TLR2 (rs4696480, rs3804100, rs1898830), TLR3(rs3775291) and TLR7(rs179008) SNPs in pregnant women who were infected with primary CMV in the first trimester, and intrauterine transmission.

	model	Genotype	Genotype Frequencies; n^a^ (%)		OR^b^ (95% CI^c^)	P-value^d^
			No transmission	Transmission		
**rs4696480**	Co-dominant	T/T	11 (30.6%)	7 (43.8%)	1	.170
		T/A	21 (58.3%)	5 (31.2%)	0.37 (0.10–1.46)	
		A/A	4 (11.1%)	4 (25%)	1.57 (0.29–8.42)	
	Dominant	T/T	11 (30.6%)	7 (43.8%)	1	.360
		T/A-A/A	25 (69.4%)	9 (56.2%)	0.57 (0.17–1.91)	
	Recessive	T/T-T/A	32 (88.9%)	12 (75%)	1	.210
		A/A	4 (11.1%)	4 (25%)	2.67 (0.57–12.40)	
**rs3804100**	-	T/T	31 (86.1%)	14 (87.5%)	1	.890
		T/C	5 (13.9%)	2 (12.5%)	0.89 (0.15–5.13)	
**rs1898830**	Co-dominant	A/A	13 (36.1%)	8 (50%)	1	.220
		A/G	18 (50%)	4 (25%)	0.36 (0.09–1.46)	
		G/G	5 (13.9%)	4 (25%)	1.30 (0.27–6.33)	
	Dominant	A/A	13 (36.1%)	8 (50%)	1	.350
		A/G-G/G	23 (63.9%)	8 (50%)	0.57 (0.17–1.86)	
	Recessive	A/A-A/G	31 (86.1%)	12 (75%)	1	.340
		G/G	5 (13.9%)	4 (25%)	2.07 (0.47–9.02)	
**rs3775291**	Co-dominant	C/C	22 (61.1%)	7 (43.8%)	1	.500
		C/T	12 (33.3%)	8 (50%)	2.10 (0.61–7.20)	
		T/T	2 (5.6%)	1 (6.2%)	1.57 (0.12–20.06)	
	Dominant	C/C	22 (61.1%)	7 (43.8%)	1	.250
		C/T-T/T	14 (38.9%)	9 (56.2%)	2.02 (0.61–6.67)	
	Recessive	C/C-C/T	34 (94.4%)	15 (93.8%)	1	.920
		T/T	2 (5.6%)	1 (6.2%)	1.13 (0.10–13.48)	
**rs179008**	Co-dominant	A/A	22 (61.1%)	10 (62.5%)	1	.840
		A/T	10 (27.8%)	5 (31.2%)	1.10 (0.30–4.07)	
		T/T	4 (11.1%)	1 (6.2%)	0.55 (0.05–5.57)	
	Dominant	A/A	22 (61.1%)	10 (62.5%)	1	.920
		A/T-T/T	14 (38.9%)	6 (37.5%)	0.94 (0.28–3.17)	
	Recessive	A/A-A/T	32 (88.9%)	15 (93.8%)	1	.570
		T/T	4 (11.1%)	1 (6.2%)	0.53 (0.05–5.19)	

^a^n, number of women in whom the onset of CMV infection was limited to the first trimester

^b^OR odd ratio

^c^CI, confidence interval

^d^P≤0.05 is considered as significant

However, in women in whom onset of infection was limited to the second trimester, a significant difference in allelic variation in TLR2 was found (Table 4); rs1898830 frequency of AA, AG and GG was 41.2%, 58.8% and 0% respectively in the transmitters group and 28.6%, 42.9% and 28.6%, respectively in the non-transmitters group. This difference was statistically significant in both the co-dominant and the recessive model of inheritance (*p* = .029 and .008, respectively) (Table 4).

**Table 4:** The distribution of genotype frequencies of TLR2 (rs4696480, rs3804100, rs1898830), TLR3 (rs3775291) and TLR7 (rs179008) SNPs in pregnant women who were infected with primary CMV in the second trimester, and intrauterine transmission.

	model	Genotype	Genotype Frequencies; n^a^ (%)		OR^b^ (95% CI^c^)	P-value^d^
			No transmission	Transmission		
**rs4696480**	Co-dominant	T/T	4 (28.6%)	5 (29.4%)	1	.410
		T/A	7 (50%)	11 (64.7%)	1.26 (0.25–6.36)	
		A/A	3 (21.4%)	1 (5.9%)	0.27 (0.02–3.65)	
	Dominant	T/T	4 (28.6%)	5 (29.4%)	1	.960
		T/A-A/A	10 (71.4%)	12 (70.6%)	0.96 (0.20–4.57)	
	Recessive	T/T-T/A	11 (78.6%)	16 (94.1%)	1	.190
		A/A	3 (21.4%)	1 (5.9%)	0.23 (0.02–2.50)	
**rs3804100**	Co-dominant	T/T	11 (78.6%)	12 (70.6%)	1	.810
		T/C	2 (14.3%)	4 (23.5%)	1.83 (0.28–12.07)	
		C/C	1 (7.1%)	1 (5.9%)	0.92 (0.05–16.50)	
	Dominant	T/T	11 (78.6%)	12 (70.6%)	1	.610
		T/C-C/C	3 (21.4%)	5 (29.4%)	1.53 (0.29–7.94)	
	Recessive	T/T-T/C	13 (92.9%)	16 (94.1%)	1	.890
		C/C	1 (7.1%)	1 (5.9%)	0.81 (0.05–14.28)	
**rs1898830**	Co-dominant	A/A	4 (28.6%)	7 (41.2%)	1	.029
		A/G	6 (42.9%)	10 (58.8%)	0.95 (0.19–4.68)	
		G/G	4 (28.6%)	0 (0%)	0.00 (0.00-NA)	
	Dominant	A/A	4 (28.6%)	7 (41.2%)	1	.460
		A/G-G/G	10 (71.4%)	10 (58.8%)	0.57 (0.13–2.58)	
	Recessive	A/A-A/G	10 (71.4%)	17 (100%)	1	.008
		G/G	4 (28.6%)	0 (0%)	0.00 (0.00-NA)	
**rs3775291**	Co-dominant	C/C	8 (57.1%)	6 (35.3%)	1	.190
		C/T	6 (42.9%)	9 (52.9%)	2.00 (0.46–8.78)	
		T/T	0 (0%)	2 (11.8%)	NA (0.00-NA)	
	Dominant	C/C	8 (57.1%)	6 (35.3%)	1	.220
		C/T-T/T	6 (42.9%)	11 (64.7%)	2.44 (0.57–10.45)	
	Recessive	C/C-C/T	14 (100%)	15 (88.2%)	1	.110
		T/T	0 (0%)	2 (11.8%)	NA (0.00-NA)	
**rs179008**	Co-dominant	A/A	9 (69.2%)	10 (58.8%)	1	.520
		A/T	4 (30.8%)	6 (35.3%)	1.35 (0.29–6.38)	
		T/T	0 (0%)	1 (5.9%)	NA (0.00-NA)	
	Dominant	A/A	9 (69.2%)	10 (58.8%)	1	.560
		A/T-T/T	4 (30.8%)	7 (41.2%)	1.57 (0.34–7.22)	
	Recessive	A/A-A/T	13 (100%)	16 (94.1%)	1	.280
		T/T	0 (0%)	1 (5.9%)	NA (0.00-NA)	

^a^n, number of women in whom the onset of CMV infection was limited to second trimester

^b^OR odd ratio

^c^CI, confidence interval

^d^P≤0.05 is considered as significant

## Discussion

In the current study we assessed correlations between specific SNPs in the TLR genes and intrauterine transmission of CMV in pregnant women with primary CMV infection. We found that the frequency of the rs1898830 polymorphism in women who were infected during the second pregnancy trimester is different in transmitters and non-transmitters. However, the clinical utilization of this finding is still premature and further studies looking at the mechanism underlying our finding are needed.

An association between the rs3804100 and rs1898830 polymorphisms in the newborn with congenital CMV infection was revealed in Japanese infants, suggesting a functional role of these SNP's in the mechanism of intrauterine transmission. It should be noted that this phenomenon was found in the newborns themselves, and it is not straightforward to apply these data directly to maternal genotypes. In addition, there is a large genetic variability between the Japanese population (JPT) and our cohort (considered as CEU). For example, in the rs3804100 SNP, the MAF in the CEU population is lower than the JPT cohort (9% vs. 25%). Similarly, in the rs1898830 the MAF is also lower in CEU as compared to JPT populations (32% vs.49%). The reduced frequency of the CC genotype of the rs3804100 SNP might obscure the difference between the women who transmitted the virus to the fetus and the women who did not.

The possible protective effect of the GG genotype of the rs1898830 SNP does not exist in women infected in the first trimester. This finding may be explained by the multi-factorial nature of maternal-fetal transmission during the various stages of pregnancy. In particular, the fact that the transmission rate is significantly lower in the first trimester might obscure a protective effect of the GG genotype of rs1898830. In addition, the expression of TLRs at the placenta appears to be regulated in a temporal and spatial course [19]. As yet no study has directly addressed the TLR2 expression level in different placental cells in relation to the three pregnancy trimesters. It was demonstrated that during the first-trimester, TLR2 is expressed by villous cytotrophoblast and extravillous trophoblast but not by syncytiotrophoblasts [20], whereas in term placentas TLR2 (and TL4) are expressed by both cytotrophoblast and syncytiotrophoblast [21]. Cooperative response of different TLRs is another aspect of the maternal-fetal interface. It appears that the TLRs composition of cells determines the response to external signals. For example, expression of TLR1 and TLR6 on trophoblasts impacts the response to TLR2 stimulation [22]. Therefore, the exact function of TLR2 in the maternal-fetal interface is still unclear. Data regarding the temporal and spatial expression of TLR2 as well as other cooperative TLRs might help to elucidate the biological protective effect of the GG genotype of the rs1898830 SNP in women infected with CMV during the second trimester.

As rs1898830 SNP is located in the TLR-2 intron, it is challenging to explain the exact mechanism of its effect. One possibility is that it might alter the expression and the functionality of TLR-2 by modifying a splicing site [23]. Alternatively, rs1898830 SNP might be associated with another SNP that impacts on the regulation of this gene. Despite the fact that the exact functional effect of the rs1898830 SNP is still unknown, it has been shown that the GG genotype for TLR2 rs1898830 has a functional effect on neonatal regulatory T cells, depending on maternal atopy [13].

## Conclusions

In this study, we chose five TLR SNPs that were shown to be associated with variations in CMV infection or cytokine response. Among women infected in the second trimester of pregnancy, a significant association was found between the rs1898830 polymorphism and intrauterine transmission.

## Supporting information

S1 Table The distribution of genotype frequencies of TLR2 (rs4696480, rs3804100, rs1898830), TLR3(rs3775291) and TLR7(rs179008) SNPs in all pregnant women who were infected with primary CMV, and intrauterine transmission. (DOCX)

## References

[1] Cheeran MC, Lokensgard JR, Schleiss MR Neuropathogenesis of congenital cytomegalovirus infection: disease mechanisms and prospects for intervention. Clin Microbiol Rev. 2009 22: 99–126, Table of Contents. doi: 10.1128/CMR.00023-08 19136436 10.1128/CMR.00023-08 PMC2620634

[2] Dollard SC, Grosse SD, Ross DS New estimates of the prevalence of neurological and sensory sequelae and mortality associated with congenital cytomegalovirus infection. Rev Med Virol. 2007 17: 355–363. doi: 10.1002/rmv.544 17542052 10.1002/rmv.544

[3] Oliver SE, Cloud GA, Sanchez PJ, Demmler GJ, Dankner W, Shelton M, et al Neurodevelopmental outcomes following ganciclovir therapy in symptomatic congenital cytomegalovirus infections involving the central nervous system. J Clin Virol. 2009 46 Suppl 4: S22–26. 19766534 10.1016/j.jcv.2009.08.012 PMC2805252

[4] Cannon MJ Congenital cytomegalovirus (CMV) epidemiology and awareness. J Clin Virol. 2009 46 Suppl 4: S6–10. 19800841 10.1016/j.jcv.2009.09.002

[5] Wang C, Zhang X, Bialek S, Cannon MJ Attribution of congenital cytomegalovirus infection to primary versus non-primary maternal infection. Clin Infect Dis. 2011 52: e11–13. doi: 10.1093/cid/ciq085 21288834 10.1093/cid/ciq085

[6] Enders G, Daiminger A, Bader U, Exler S, Enders M Intrauterine transmission and clinical outcome of 248 pregnancies with primary cytomegalovirus infection in relation to gestational age. J Clin Virol. 2011 52: 244–246. doi: 10.1016/j.jcv.2011.07.005 21820954 10.1016/j.jcv.2011.07.005

[7] De Nardo D Toll-like receptors: Activation, signalling and transcriptional modulation. Cytokine. 2015 74: 181–189. doi: 10.1016/j.cyto.2015.02.025 25846205 10.1016/j.cyto.2015.02.025

[8] Wujcicka W, Wilczynski J, Nowakowska D Alterations in TLRs as new molecular markers of congenital infections with Human cytomegalovirus? Pathog Dis. 2013 70: 3–16. doi: 10.1111/2049-632X.12083 23929630 10.1111/2049-632X.12083

[9] Arav-Boger R, Wojcik GL, Duggal P, Ingersoll RG, Beaty T, Pass RF, et al Polymorphisms in Toll-like receptor genes influence antibody responses to cytomegalovirus glycoprotein B vaccine. BMC Res Notes. 2012 5: 140 doi: 10.1186/1756-0500-5-140 22414065 10.1186/1756-0500-5-140 PMC3317442

[10] Ducloux D, Deschamps M, Yannaraki M, Ferrand C, Bamoulid J, Saas P, et al Relevance of Toll-like receptor-4 polymorphisms in renal transplantation. Kidney Int. 2005 67: 2454–2461. doi: 10.1111/j.1523-1755.2005.00354.x 15882292 10.1111/j.1523-1755.2005.00354.x

[11] Nahum A, Dadi H, Bates A, Roifman CM The biological significance of TLR3 variant, L412F, in conferring susceptibility to cutaneous candidiasis, CMV and autoimmunity. Autoimmun Rev. 2012 11: 341–347. doi: 10.1016/j.autrev.2011.10.007 22024499 10.1016/j.autrev.2011.10.007

[12] Taniguchi R, Koyano S, Suzutani T, Goishi K, Ito Y, Morioka I, et al Polymorphisms in TLR-2 are associated with congenital cytomegalovirus (CMV) infection but not with congenital CMV disease. Int J Infect Dis. 2013 17: e1092–1097. doi: 10.1016/j.ijid.2013.06.004 23906542 10.1016/j.ijid.2013.06.004

[13] Liu J, Radler D, Illi S, Klucker E, Turan E, von Mutius E, et al TLR2 polymorphisms influence neonatal regulatory T cells depending on maternal atopy. Allergy. 2011 66: 1020–1029. doi: 10.1111/j.1398-9995.2011.02573.x 21371045 10.1111/j.1398-9995.2011.02573.x

[14] Kijpittayarit S, Eid AJ, Brown RA, Paya CV, Razonable RR Relationship between Toll-like receptor 2 polymorphism and cytomegalovirus disease after liver transplantation. Clin Infect Dis. 2007 44: 1315–1320. doi: 10.1086/514339 17443468 10.1086/514339

[15] Revello MG, Gerna G Diagnosis and management of human cytomegalovirus infection in the mother, fetus, and newborn infant. Clin Microbiol Rev. 2002 15: 680–715. doi: 10.1128/CMR.15.4.680-715.2002 12364375 10.1128/CMR.15.4.680-715.2002 PMC126858

[16] Sole X, Guino E, Valls J, Iniesta R, Moreno V SNPStats: a web tool for the analysis of association studies. Bioinformatics. 2006 22: 1928–1929. doi: 10.1093/bioinformatics/btl268 16720584 10.1093/bioinformatics/btl268

[17] Revello MG, Zavattoni M, Sarasini A, Percivalle E, Simoncini L, Gerna G Human cytomegalovirus in blood of immunocompetent persons during primary infection: prognostic implications for pregnancy. J Infect Dis. 1998 177: 1170–1175. 9592999 10.1086/515277

[18] Stagno S, Pass RF, Cloud G, Britt WJ, Henderson RE, Walton PD, et al Primary cytomegalovirus infection in pregnancy. Incidence, transmission to fetus, and clinical outcome. JAMA. 1986 256: 1904–1908. 3020264

[19] Koga K, Izumi G, Mor G, Fujii T, Osuga Y Toll-like receptors at the maternal-fetal interface in normal pregnancy and pregnancy complications. Am J Reprod Immunol. 2014 72: 192–205. doi: 10.1111/aji.12258 24754320 10.1111/aji.12258

[20] Abrahams VM, Bole-Aldo P, Kim YM, Straszewski-Chavez SL, Chaiworapongsa T, Romero R, et al Divergent trophoblast responses to bacterial products mediated by TLRs. J Immunol. 2004 173: 4286–4296. 15383557 10.4049/jimmunol.173.7.4286

[21] Mitsunari M, Yoshida S, Shoji T, Tsukihara S, Iwabe T, Harada T, et al Macrophage-activating lipopeptide-2 induces cyclooxygenase-2 and prostaglandin E(2) via toll-like receptor 2 in human placental trophoblast cells. J Reprod Immunol. 2006 72: 46–59. doi: 10.1016/j.jri.2006.02.003 16600383 10.1016/j.jri.2006.02.003

[22] Abrahams VM, Aldo PB, Murphy SP, Visintin I, Koga K, Wilson G, et al TLR6 modulates first trimester trophoblast responses to peptidoglycan. J Immunol. 2008 180: 6035–6043. 18424724 10.4049/jimmunol.180.9.6035 PMC3025812

[23] Wang GS, Cooper TA Splicing in disease: disruption of the splicing code and the decoding machinery. Nat Rev Genet. 2007 8: 749–761. doi: 10.1038/nrg2164 17726481 10.1038/nrg2164

