# Supplementary material for: Association of toll-like receptors polymorphism and intrauterine transmission of cytomegalovirus
Source: PLoS One. 2017 Dec 21;12(12):e0189921. doi: 10.1371/journal.pone.0189921 (PMC5739442; doi:10.1371/journal.pone.0189921)
Supplement: S1 Table — (DOCX) [file pone.0189921.s001.docx]

**S1 Table**. The distribution of genotype frequencies of TLR2 (rs4696480, rs3804100, rs1898830), TLR3(rs3775291) and TLR7(rs179008) SNPs in all pregnant women who were infected with primary CMV, and intrauterine transmission.

|  | model | Genotype | Genotype Frequencies; n^a^ (%) | | OR^b^ (95% CI^c^) | P-value |
| --- | --- | --- | --- | --- | --- | --- |
|  |  |  | No transmission | Transmission |  |  |
| rs4696480 | Co-dominant | T/T | 15 (30%) | 12 (36.4%) | 1 | .79 |
|  |  | T/A | 28 (56%) | 16 (48.5%) | 0.71 (0.27-1.90) |  |
|  |  | A/A | 7 (14%) | 5 (15.2%) | 0.89 (0.23-3.53) |  |
|  | Dominant | T/T | 15 (30%) | 12 (36.4%) | 1 | .55 |
|  |  | T/A-A/A | 35 (70%) | 21 (63.6%) | 0.75 (0.30-1.91) |  |
|  | Recessive | T/T-T/A | 43 (86%) | 28 (84.8%) | 1 | .88 |
|  |  | A/A | 7 (14%) | 5 (15.2%) | 1.10 (0.32-3.80) |  |
| rs3804100 | Co-dominant | T/T | 42 (84%) | 26 (78.8%) | 1 | .83 |
|  |  | T/C | 7 (14%) | 6 (18.2%) | 1.38 (0.42-4.57) |  |
|  |  | C/C | 1 (2%) | 1 (3%) | 1.62 (0.10-26.96) |  |
|  | Dominant | T/T | 42 (84%) | 26 (78.8%) | 1 | .55 |
|  |  | T/C-C/C | 8 (16%) | 7 (21.2%) | 1.41 (0.46-4.36) |  |
|  | Recessive | T/T-T/C | 49 (98%) | 32 (97%) | 1 | .77 |
|  |  | C/C | 1 (2%) | 1 (3%) | 1.53 (0.09-25.37) |  |
| rs1898830 | Co-dominant | A/A | 17 (34%) | 15 (45.5%) | 1 | .53 |
|  |  | A/G | 24 (48%) | 14 (42.4%) | 0.66 (0.25-1.72) |  |
|  |  | G/G | 9 (18%) | 4 (12.1%) | 0.50 (0.13-1.98) |  |
|  | Dominant | A/A | 17 (34%) | 15 (45.5%) | 1 | .30 |
|  |  | A/G-G/G | 33 (66%) | 18 (54.5%) | 0.62 (0.25-1.52) |  |
|  | Recessive | A/A-A/G | 41 (82%) | 29 (87.9%) | 1 | .46 |
|  |  | G/G | 9 (18%) | 4 (12.1%) | 0.63 (0.18-2.24) |  |
| rs3775291 | Co-dominant | C/C | 30 (60%) | 13 (39.4%) | 1 | .16 |
|  |  | C/T | 18 (36%) | 17 (51.5%) | 2.18 (0.86-5.52) |  |
|  |  | T/T | 2 (4%) | 3 (9.1%) | 3.46 (0.52-23.23) |  |
|  | Dominant | C/C | 30 (60%) | 13 (39.4%) | 1 | .07 |
|  |  | C/T-T/T | 20 (40%) | 20 (60.6%) | 2.31 (0.94-5.67) |  |
|  | Recessive | C/C-C/T | 48 (96%) | 30 (90.9%) | 1 | .35 |
|  |  | T/T | 2 (4%) | 3 (9.1%) | 2.40 (0.38-15.21) |  |
| rs179008 | Co-dominant | A/A | 31 (63.3%) | 20 (60.6%) | 1 | .87 |
|  |  | A/T | 14 (28.6%) | 11 (33.3%) | 1.22 (0.46-3.21) |  |
|  |  | T/T | 4 (8.2%) | 2 (6.1%) | 0.78 (0.13-4.63) |  |
|  | Dominant | A/A | 31 (63.3%) | 20 (60.6%) | 1 | .81 |
|  |  | A/T-T/T | 18 (36.7%) | 13 (39.4%) | 1.12 (0.45-2.78) |  |
|  | Recessive | A/A-A/T | 45 (91.8%) | 31 (93.9%) | 1 | .72 |
|  |  | T/T | 4 (8.2%) | 2 (6.1%) | 0.73 (0.13-4.21) |  |

^a^n, number of women;

^b^OR, odd ratio;

^c^CI, confidence interval;

^d^P≤0.05 is considered as significant.
